# Supplementary material for: Long-Chain Acyl Coenzyme A Dehydrogenase, a Key Player in Metabolic Rewiring/Invasiveness in Experimental Tumors and Human Mesothelioma Cell Lines
Source: Cancers (Basel). 2023 Jun 3;15(11):3044. doi: 10.3390/cancers15113044 (PMC10252348; doi:10.3390/cancers15113044)
Supplement: Supplementary file 1 [file cancers-15-03044-s001.zip › Supplementary Table S1.pdf]

Supplementary Table S1. Primers for gene expression analysis.

Gene	Forward sequence	Reverse sequence
**ACADL**	TGCAATAGCAATGACAGAGCC	CGCAACTACAATCACAACATCAC
**ALDH7A1**	ATGGCAAGCCCTATGTCATCT	CCGTGGTACTTATCAGCCCA
**ATP5H**	GCTGGGCGAAAACTTGCTCTA	CCAGTCGATAGCTGGTGGATT
**ATPO**	ATTGAAGGTCGCTATGCCACA	GCTTTTCACTTTAATGGAACGCT
**BCAT2**	CGCTCCTGTTCGTCATTCTCT	CCCACCTAACTTGTAGTTGCC
**MTCO2**	ACAGATGCAATTCCCGGACGTCTA	GGCATGAAACTGTGGTTTGCTCCA
**COX5B**	TGTGAAGAGGACAATACCAGCG	CCAGCTTGTAATGGGCTCCAC
**COX6C2**	TGTTGGCTGCTGCGTCACATTC	CAGAATCTTCCAGGTCCTCGCTCC
**TUFM**	GGGGCTAAGTTCAAGAAGTACG	CACATGAGCCGCATTGATGG
**HSD17B10**	TGGCGGTAATAACCGGAGGA	ACAGTTGACAGCTACATCCACA
**IDH3A**	CCCGCGTGGATCTCTAAGG	AATTTCTGGGCCAATACCATCTC
**IDH3B**	GAGCCAAGTCTCAGCGGATT	GGGCATCACAAGCACATCAAA
**AK2**	GCAGAACCCGAGTATCCTAAAGG	TTCCCAGCATCCATAGTTGCC
**MDH2**	TCGGCCCAGAACAATGCTAAA	GCGGCTTTGGTCTCGATGT
**MYG1**	ACAATGGCACCTTCCACTGCGA	ACCACGATGTCACAGGAAGCGA
**OAT**	TGGACCATTTATGCCGGGATT	GCTTCACCCTGAATTGGTTCT
**PHB**	GACCACGTAATGTGCCAGTCA	CATCATAGTCCTCTCCGATGCT
**PHB2**	GTGCGCGAATCTGTGTTCAC	GATAATGGGGTACTGGAACCAAG
**SSBP1**	TGAGTCCGAAACAACTACCAGT	CCTGATCGCCACATCTCATTAG
**TRAP1**	AGGACGACTGTTCAGCACG	CCGGGCAACAATGTCCAAAAG
**ACADS**	GATGGCAAATGTAGACCCTACC	AAGGCCCGGAGTATCACGA
**ACO2**	GAGCGAGGCAAGTCGTACC	GGCTTCAATCAGATGGTCACAG
**CS**	TGCTTCCTCCACGAATTTGAAA	CCACCATACATCATGTCCACAG
**DECR1**	TCTTCAAAAAGCGATGCTACCA	CTATCACGCACTGAGCACCT
**GSTP1**	CCCTACACCGTGGTCTATTTCC	CAGGAGGCTTTGAGTGAGC
**HCDH**	ATATGCCGCAATTTTACAGGGT	ACCTGCAATAAAGCAGCCTGG
**IVD**	GCTGACCTGTTGAGTGAGGC	TCGGTGAGCGTCTTGGTCTTA
**MGST1**	ATGACAGAGTAGAACGTGTACGC	TACAGGAGGCCAATTCCAAGA
**DLST**	GAACTGCCCTCTAGGGAGAC	AACCTTCCTGCTGTTAGGGTA
**PRDX3**	GGCCTGTCTGAGTGTTAATGATG	GGAGCCGAACCTTGCCTTC
**RMDN3**	CCAGCGATGGAAACGGACC	GGGATCTGAAGTCTGCGTATAGT
**S100A10**	GGCTACTTAACAAAGGAGGACC	GAGGCCCGCAATTAGGGAAA
**SUOX**	ACTCAAGTCAATCCCCTCAAGG	GCTGGAGTTATCACCAGAGAAGG
**MPST**	CGCCGTGTCACTGCTTGAT	CAGGTTCAATGCCGTCTCG
**TST**	ACTTCACCAGACCAAGGAGAT	CCGACAGCATTTCCACAATTTT
**TIMM9**	AGAGACCTGCTTTTTGGACTGTG	CCTGAAATCTCATGGATATTCTTTG
