# Supplementary figures and images for: Long-Chain Acyl Coenzyme A Dehydrogenase, a Key Player in Metabolic Rewiring/Invasiveness in Experimental Tumors and Human Mesothelioma Cell Lines
Source: Cancers (Basel). 2023 Jun 3;15(11):3044. doi: 10.3390/cancers15113044 (PMC10252348; doi:10.3390/cancers15113044)

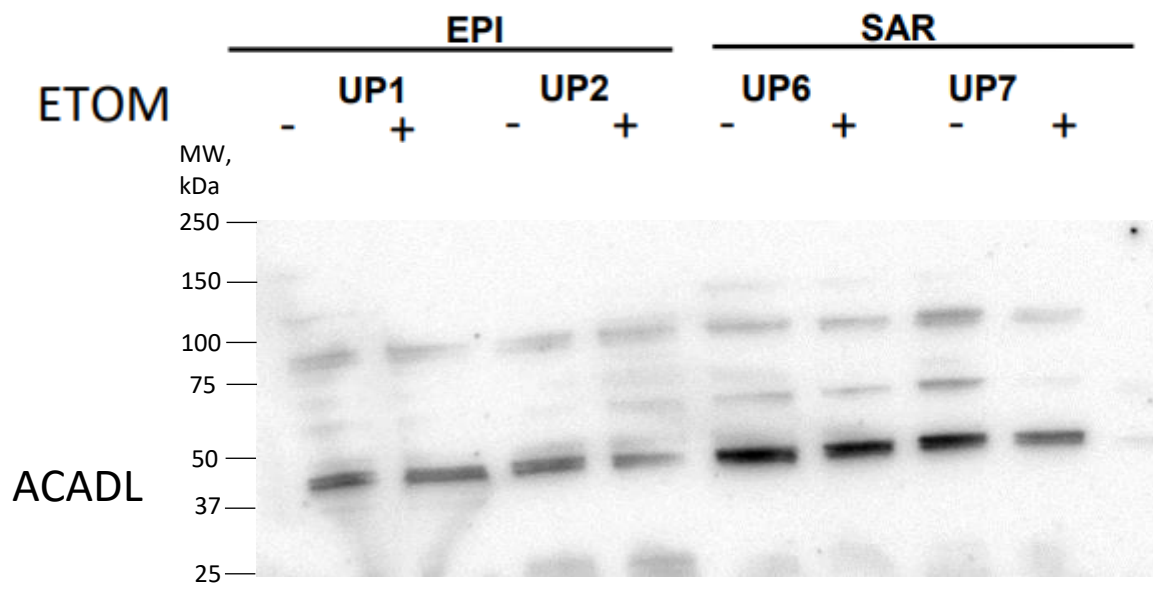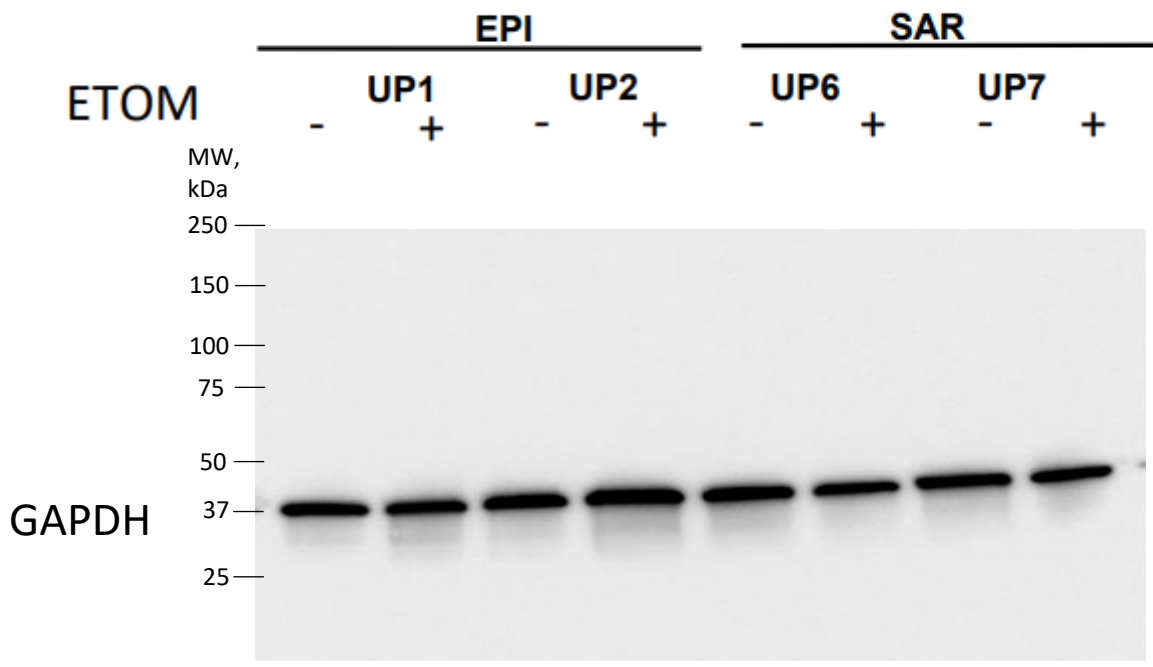

Supplement: Supplementary file 1 [file cancers-15-03044-s001.zip › Supplementary Figure S1.pdf]
